# Sexual Dimorphism in the Immunometabolic Role of Gpr183 in Mice

**DOI:** 10.1210/jendso/bvae188

**Published:** 2024-10-29

**Authors:** Liv von Voss, Tulika Arora, Juliana Assis, Katharina B Kuentzel, Kristine N Arfelt, Mark K Nøhr, Trisha J Grevengoed, Manimozhiyan Arumugam, Thomas Mandrup-Poulsen, Mette M Rosenkilde

**Affiliations:** Molecular and Translational Pharmacology, Department of Biomedical Sciences, University of Copenhagen, DK 2200 Copenhagen, Denmark; Novo Nordisk Foundation Center for Basic Metabolic Research and Medical Sciences, University of Copenhagen, DK 2200 Copenhagen, Denmark; Novo Nordisk Foundation Center for Basic Metabolic Research and Medical Sciences, University of Copenhagen, DK 2200 Copenhagen, Denmark; National Bioinformatics Infrastructure Sweden, Science for Life Laboratory, Department of Immunotechnology, Lund University, SE 223 63 Lund, Sweden; Molecular and Translational Pharmacology, Department of Biomedical Sciences, University of Copenhagen, DK 2200 Copenhagen, Denmark; Molecular and Translational Pharmacology, Department of Biomedical Sciences, University of Copenhagen, DK 2200 Copenhagen, Denmark; Molecular and Translational Pharmacology, Department of Biomedical Sciences, University of Copenhagen, DK 2200 Copenhagen, Denmark; Molecular and Translational Pharmacology, Department of Biomedical Sciences, University of Copenhagen, DK 2200 Copenhagen, Denmark; Novo Nordisk Foundation Center for Basic Metabolic Research and Medical Sciences, University of Copenhagen, DK 2200 Copenhagen, Denmark; Molecular and Translational Pharmacology, Department of Biomedical Sciences, University of Copenhagen, DK 2200 Copenhagen, Denmark; Molecular and Translational Pharmacology, Department of Biomedical Sciences, University of Copenhagen, DK 2200 Copenhagen, Denmark

**Keywords:** GPR183, G protein–coupled receptor, sexual dimorphism, oxysterols, Gpr183 knockout mouse, high-fat, high-sucrose diet

## Abstract

**Context:**

Excessive eating and intake of a Western diet negatively affect the intestinal immune system, resulting in compromised glucose homeostasis and lower gut bacterial diversity. The G protein–coupled receptor GPR183 regulates immune cell migration and intestinal immune response and has been associated with tuberculosis, type 1 diabetes, and inflammatory bowel diseases.

**Objective:**

We hypothesized that with these implications, GPR183 has an important immunometabolic role and investigated this using a global Gpr183 knockout mouse model.

**Methods:**

Wild-type (WT) and *Gpr183*-deficient (Gpr183^–/–^) mice were fed a high-fat, high-sucrose diet (HFSD) for 15 weeks. We investigated changes in weight, body composition, fecal immunoglobulin A (IgA) levels, fecal microbiome, and glucose tolerance before and after the diet. Macrophage infiltration into visceral fat was determined by flow cytometry, and hepatic gene expression was measured.

**Results:**

A sexual dimorphism was discovered, whereby female Gpr183^–/–^ mice showed adverse metabolic outcomes compared to WT counterparts with inferior glucose tolerance, lower fecal IgA levels, and increased macrophage infiltration in visceral fat. In contrast, male Gpr183^–/–^ mice had significantly lower fasting blood glucose after diet than male WT mice. Liver gene expression showed reduced inflammation and macrophage markers in Gpr183^–/–^ livers, regardless of sex, while the pancreatic islet area did not differ between the groups. No conclusive differences were found after microbiome sequencing.

**Conclusion:**

Gpr183 maintains metabolic homeostasis in female but not in male mice independent of diet. If confirmed in humans, future therapy targeting GPR183 should consider this sexual dimorphism.

The G protein-coupled receptor 183 (GPR183) is highly expressed in immune cells, especially B cells [1-4], and is important for B-cell maturation and localization within secondary lymphoid organs [2, 3, 5, 6]. It belongs to class A G protein-coupled receptors (GPCRs) and signals via Gαi that, together with its strong arrestin recruitment, regulate cell migration [1, 7]. Its deorphanization in 2011 revealed oxysterols, for example, 7α,25-dihydroxycholesterol (7α,25-OHC) and 7α,27-dihydroxycholesterol (7α,27-OHC) as endogenous ligands, with 7α,25-OHC being the most potent agonist [5, 8, 9]. Oxysterols are oxidized products derived from cholesterol associated with several immunometabolic conditions such as obesity, diabetes, and metabolic syndrome [10-14]. In people with diabetes, plasma levels of the preproducts of 7α,25-OHC and 7α,27-OHC; 25-hydroxycholesterol (25-OHC), 27-hydroxycholesterol (27-OHC), together with visceral adipose tissue (VAT)-expressed cholesterol 25-hydroxylase (CH25H), are increased compared to individuals without diabetes [12-14]. In females with metabolic syndrome, serum 25-OHC, 7α- and 7β-dihydroxycholesterol are higher compared to healthy controls, whereas no differences are seen in males with metabolic syndrome, showing these oxysterol differences to be sex dependent [11].

GPR183 was recently linked to inflammatory bowel diseases [15]. Supporting its role in intestinal homeostasis, Gpr183-oxysterol interaction is required for innate lymphoid cells, for example, group 3 innate lymphoid cells' (ILC3s) migration to colonic lymphoid structures in mice [16, 17]. ILC3s are known for their dominant role in intestinal barrier integrity [18]. It is further known that intestinal differentiated B cells, also known as intestinal plasma cells, are important for immune defense and the production of immunoglobulin A (IgA). Gpr183 has also been identified as central for the B2 lymphocytes' distribution in the omentum and the peritoneal [19]. Finally, another link to intestinal homeostasis for GPR183 was recently established by identifying the microbial metabolite, lauroyl tryptamine, as an antagonist for GPR183 [20].

Obesity is a risk factor for impaired glucose tolerance and chronic systemic low-grade inflammation [21]. Recently, the gut microbiome and the intestinal immune response have become more recognized as important factors in obesity and diabetes [22, 23]. An important finding is that the colonic inflammatory response to a high-fat diet occurs before the inflammatory response within the AT and most likely initiates systemic low-grade inflammation and impaired glucose tolerance within the AT [24]. Despite the altered levels of oxysterols in metabolic diseases and our current insight into the role of GPR183 in the intestinal immune response, combined with a recently proven expression of GPR183 in rat and human islets [25], surprisingly little is known about GPR183 in metabolic diseases.

We hypothesize that Gpr183 plays a role in the crosstalk between intestinal and AT inflammation and thereby affects glucose homeostasis. In this study, we established a global *Gpr183*-deficient (Gpr183^–/–^) mouse model and challenged it metabolically through a high-fat, high-sucrose diet (HFSD). We examined body weight and composition, fecal microbiome, and glucose tolerance. Finally, we measured fecal IgA levels before and after HFSD and investigated VAT macrophage infiltration on termination of the study. We found that female Gpr183^–/–^ mice had lower fecal IgA levels compared to wild-type (WT) before and after HFSD, together with increased macrophage infiltration in VAT and impaired glucose tolerance, not compensated by increased insulin secretion to WT. Male Gpr183^–/–^ had a significantly lower body weight before HFSD and no differences in glucose tolerance or VAT macrophage infiltration. These data position Gpr183 as a potential drug target when combating metabolic dysfunction and its comorbidities. Our study emphasizes the importance of dual-sex studies based on pronounced sexual differences in the role of Gpr183 in metabolism.

## Material and Methods

### Animals

Animal studies were performed under the instructions of the ARRIVE 2.0 guidelines [26]. A global constitutive Gpr183 knockout (Gpr183^–/–^) mouse was generated by an intragenic deletion at Lexicon Pharmaceuticals, Gpr183^tm1Lex^, and bred on a C57BL6Jtacbom background at the University of Copenhagen. Together with WT C57BL6Jtacbom female and male mice, they were bred and housed at the University of Copenhagen. All mice were weekly weighted and body composition biweekly assessed by echo magnetic resonance imaging (LF90II body composition analyzer, Bruker), and at age 11 to 13 weeks, all animals were introduced to a 58% kcal fat and 26% kcal high-sucrose diet (D12331i, Research Diets Inc). All mice were group-housed in individually ventilated cages with a 12-hour light cycle with ad libitum access to food and water. Group sizes were selected from an explorative perspective and based on availability, and no power calculations were performed [27-29]; group sizes are provided in figure legends. All experiments were conducted in accordance with institutional guidelines (EMED, University of Copenhagen) and approved by the Animal Experiments Inspectorate, Danish Ministry of Environment and Food (license No. 2018-15-0201-01442).

### Oral Glucose Tolerance Test

Mice were fasted for 4 hours with free access to water. We collected blood glucose (BG) samples with a glucometer (Contour Next, Bayer) and insulin measurements from the tail vein. During the experiment, mice stayed in their respective groups and were given orally 0.1 mL/10 g body weight of 0.15 g/mL D-glucose solution. Blood samples were collected at 0, 15, 30, 60, and 120 minutes. For insulin measurements, an approximately 75-μL blood sample was collected at 0 and 15 minutes using EDTA-coated glass capillaries (Vitrex Medical). Whole-blood samples for insulin samples were spun, and plasma was isolated for insulin level determination by ultrasensitive insulin enzyme-linked immunosorbent assay (ELISA) kit (Mercodia catalog No. 10-1249-01, RRID:AB_3101981) according to the manufacturer's protocol; results were read at 450 nm (Perkin Elmer, Envision, 2104), and concentration was calculated according to the protocol description.

### Hepatic Gene Expression

Total RNA was isolated from the livers with Trizol reagent (Thermo Scientific) according to the manufacturer's guidelines. Afterward, 2 µg of RNA was reverse-transcribed using the High Capacity complementary DNA (cDNA) Reverse Transcription Kit (Applied Biosciences). A total of 6 ng of complementary DNA was used for quantitative real-time polymerase chain reaction (PCR) with the PowerUp SYBR Green Master Mix for qPCR (Thermo Scientific). All samples were analyzed in duplicate and normalized to *Rpl13a1* as a housekeeping gene. For the real-time PCR analysis, the 2^−ΔΔCT^ method was used (primer sequences can be found in Supplemental Fig. 1 [30]).

### Flow Cytometry

VAT was collected in Dulbecco’s modified Eagle’s medium + 3% fetal calf serum. C-tubes (GentleMACS) were used to disrupt the VAT, and after that, collagenase II (10 mg/mL) was added for digestion for 20 to 30 minutes. After digestion, cells were washed and stained for leukocytes (CD45), murine macrophages (F4/80), pan macrophages (CD11b) and M1 (CD38), and fixed overnight. LSRFortessa 3 laser (BD Bioscience) was used for analysis, and flow cytometry results were analyzed using FlowJo v10.8.1 Software (BD Life Sciences).

### Immunoglobulin A Enzyme-linked Immunosorbent Assay

Fecal samples were collected before HFSD (age 8-10 weeks) and 10 weeks after HFSD start (age 19-21 weeks). The fecal collection was manually collected in the morning for the best success rate; some animals did not deliver, hence the difference in group size. Samples were snap-frozen and stored at −80 °C before analysis. Samples were weighed and homogenized in a 1% protease inhibitor cocktail. The supernatant was diluted 1000 times in assay buffer before analysis. IgA ELISA kit plates (Thermo Fisher Scientific catalog No. 88-50450-22, RRID:AB_2574850) were used and coated for 24 hours at 4 °C before the assay. Results were read at 450 nM on Envision (Envision 2104, Perkin Elmer), and concentrations were calculated according to the protocol description.

### Microbiome 16S Ribosomal RNA Gene Amplicon Sequencing and Data Analysis

One to two fecal pellets were collected before (age 9-11 weeks) and after (age 18-20 weeks) HFSD. The fecal collection was manually collected in the morning for the best success rate; some animals did not deliver, hence the difference in group size. NucleoSpin Soil DNA kit (Macherey-Nagel) was used to extract microbial genomic DNA from the fecal samples. The V4-variable region of 16S ribosomal RNA (rRNA) genes was sequenced using Illumina HiSeq 2500 (by BGI-Shenzhen), producing 2 × 250 bp paired-end reads. In total, amplicon sequencing data from 117 samples were processed. DADA2 v1.16 R package [31] was used with default parameters for the initial processing of reads. Primers were removed from raw reads using cutadapt v4.2 software [32], and reads were filtered and trimmed using “truncLen = c(220,180), trimLeft = c(10,10), maxN = 0, maxEE = c(2,2), and truncQ = 2.”

To reduce the error rate of the high-sequence throughput, 80 000 high-quality filtered and trimmed reads were randomly selected to continue the analysis. The error-rate-learning step was performed. Reads were dereplicated and merged, with the minimum overlap parameter set to 16 bp. Chimeras were identified and removed. An extra downstream analysis was performed using the LULU package [33] to reduce the number of erroneous amplicon sequence variants (ASVs), leaving 399 unique ASVs. This resulted in a final set of 3 897 712 paired-end reads with 33 313 reads per sample.

Taxonomy was assigned at the species level with the help of the SILVA database [34] release 138, using silva_nr_v138_train_set.fa.gz for annotation until the genus level, and silva_species_assignment_v138.fa for species-level resolution of taxonomic assignment. Alpha diversity (ASV richness and Shannon diversity) was estimated using the phyloseq package [35] v1.30.0. Associations of alpha diversity with each of the 3 factors—sex (females vs males), genotype (Gpr183^–/–^ vs WT), and HFSD treatment (before vs after treatment)—were investigated after controlling for the other 2 factors using the Wilcoxon rank sum test implemented in the “coin” package [36] v1.4-2. *P* values were adjusted for multiple testing using the false discovery rate (FDR) procedure and were considered statistically significant when FDR was below .05. Alpha diversity (ASV richness and Shannon diversity) was estimated using the phyloseq package [35] v1.30.0. Associations of alpha diversity with each of the 3 factors—sex (females vs males), genotype (Gpr183^–/–^ vs WT), and HFSD treatment (before vs after treatment)—were investigated after controlling for the other 2 factors using the Wilcoxon rank sum test implemented in the “coin” package [36] v1.4-2. *P* values were adjusted for multiple testing using the FDR procedure and were considered statistically significant when FDR was below .05. ASV differential abundance tests between groups were conducted using the negative binomial Wald test from DESeq2 package [37] v1.36.0. *P* values were automatically adjusted for multiple testing using the FDR procedure by DESeq2. To investigate whether our data contained closely related species *to Eubacterium rectale* DSM 17629 (producer of lauroyl tryptamine), we performed a BLAST search against the 16S rRNA gene sequence from this strain. Two ASVs (*Roseburia* [93] and *Roseburia* [376]) showed more than 97% similarity (97.6% and 97.2%, respectively), suggesting that they could belong to the same species as *E rectale* DSM 17629. To investigate whether our data contained closely related species to *E rectale* DSM 17629 (producer of lauroyl tryptamine), we performed a BLAST search against the 16S rRNA gene sequence from this strain. Two ASVs (*Roseburia* [93] and *Roseburia* [376]) showed more than 97% similarity (97.6% and 97.2%, respectively), suggesting that they could belong to the same species as *E rectale* DSM 17629. Sequencing reads have been deposited at the NCBI Short Read Archive under BioProject identifier PRJNA1031396 [38].

### Histology and Insulin Staining and Measurement

Pancreatic tissue from male and female mice WT and Gpr183^–/–^ mice, euthanized after 17 weeks of HFSD, were cut in slide sections of 4 µm and dewaxed through Tissue-Clear, alcohol, and tap water. Next, 10 minutes of preincubation in 2% bovine serum albumin was performed before overnight incubation at 4 °C with a primary antibody. The following antibody was used: insulin 2006-4 “in-house” antibody (diluted 1:15.000), from the Jens Juul Holst laboratory, University of Copenhagen. The next day, the sections were incubated for 40 minutes with a secondary antibody to amplify the reaction. Biotinylated secondary immunoglobulins were used (Goat antiguinea pig, biotinylated, BA-7000, Vector Laboratories, diluted 1:200). Next, hydrogen peroxide 3% was added to block endogenous peroxidase. The third layer consisted of a preformed avidin and biotinylated horseradish peroxidase macromolecular complex (ABC) (code No. PK-4000, Vector Laboratories) and incubated for 30 minutes. The reaction was developed using DAB (diaminobenzidine; code No. SK-4100) for 15 minutes. Last, counterstaining with Mayer's hematoxylin was performed.

Stained sections were scanned by Axioscan 7 (Zeiss) and whole-slide analyzed by QuPath 0.4.2 software for percentage insulin determination.

### Statistical Analysis

The quantitative data obtained by the experiments were analyzed by inferential statistical analysis in accordance with the experimental protocols and the nature of the data. For multiple comparisons with 1 or 2 independent variables, analysis of variance with the Tukey multiple comparisons test was performed. Microbiome 16S rRNA gene data are shown as box and whiskers with Tukey whiskers. For gene expression data, an unpaired *t* test was performed for each gene within the sexes. The individual area under the curve (iAUC) was calculated from each plot and compared by unpaired *t* test. All insulin data were tested for outliers, but none were detected. Results are expressed as mean ± SEM. A *P* level of .05 or less was considered statistically significant. Analyses were performed using GraphPad Prism 9.

## Results

### Gpr183 Affects Fat Mass Gain and Baseline Body Weight in a Sex-Diverse Manner

**Figure 1.**
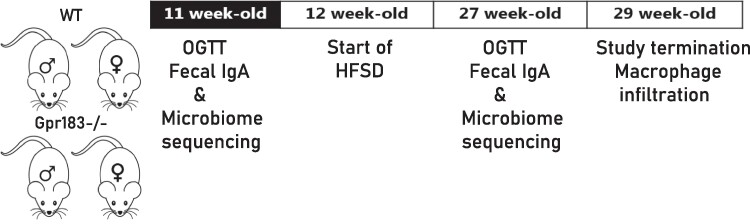
Dietary and intervention plan. Chow-fed wild-type (WT) male and female mice together with mice globally lacking Gpr183 (Gpr183^–/–^) male and female at age 11 weeks were challenged with oral glucose tolerance test (OGTT) and fecal immunoglobulin A levels and microbiome analyzed. At age 12 weeks, a high-fat, high-sucrose diet (HFSD) was initiated for 15 weeks before a final OGTT, and fecal IgA levels and microbiome were analyzed at age 27 weeks. Mice were euthanized at age 29 weeks and macrophage infiltration in the adipose tissue was investigated.

**Figure 2.**
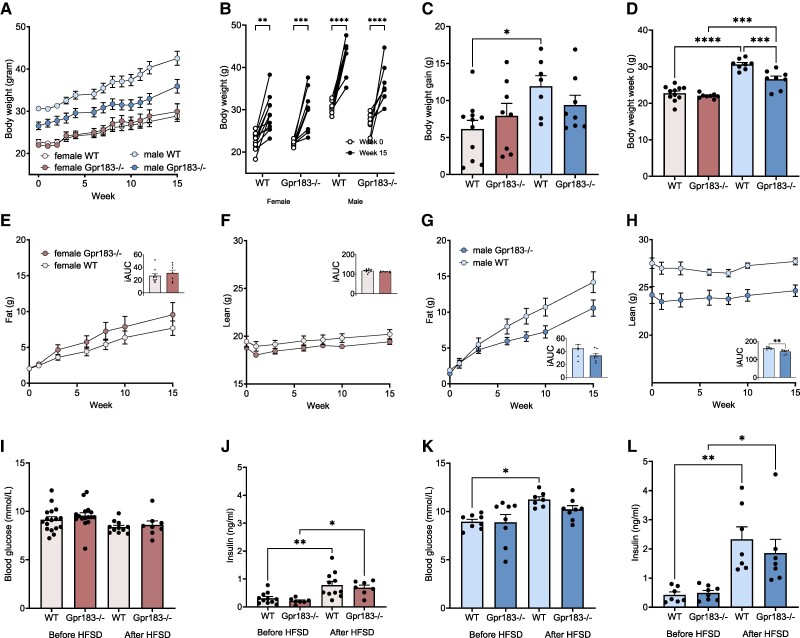
Body weight and composition development, fasting blood glucose, and insulin in wild-type (WT) and mice globally lacking Gpr183 (Gpr183^–/–^). A, Development of body weight during the study. B and C, Body weight gain at week 15. D, Body weight at diet start, week 0. E, Fat mass (g) in female mice, also represented by individual area under the curve (iAUC). F, Lean mass (g) in female mice, also represented by iAUC. G, Fat mass (g) in male mice, also represented by iAUC. H, Lean mass (g) in male mice, also represented by iAUC. I, Blood glucose levels before and after high-fat, high-sucrose diet (HFSD) in female mice. J, Insulin levels before and after HFSD in female mice. K, Blood glucose levels before and after HFSD in male mice. L, Insulin levels before and after HFSD in male mice. Insulin data were obtained from oral glucose tolerance test at time 0, before, and after HFSD. Number of female mice: WT n = 11 and Gpr183^–/–^ n = 8. Number of male mice: WT n = 8 and Gpr183^–/–^ n = 8. Statistical differences were calculated by 1-way analysis of variance (ANOVA) Tukey multiple comparisons test or paired 2-way ANOVA Sidák multiple comparisons test, and iAUC was analyzed by unpaired *t* test. All data shown as mean ± SEM, except B is shown as individuals. *P less than .05; **P less than .01; ****P less than .0001.

To investigate the role of Gpr183 on body weight and composition, we caged 11- to 13-week-old Gpr183^–/–^ and WT mice according to sex and fed them an HFSD for 17 weeks (Fig. 1). Mice were weighed weekly, and biweekly fat and lean composition measures were taken using echo magnetic resonance imaging. Irrespective of genotype, all mice increased in body weight during the HFSD (Fig. 2A and 2B). As expected from the literature [39], WT males gained significantly more weight than WT females on 15 weeks of HFSD (Fig. 2C). However, no difference was seen between female Gpr183^–/–^ and male Gpr183^–/–^ see (Fig. 2C). Before initiation of HFSD (week 0), male Gpr183^–/–^ mice had a significantly lower body weight compared to WT male mice at baseline, whereas female WT and Gpr183^–/–^ mice showed no difference in body weight at this time (Fig. 2D). Both female groups increased in fat mass during the study, with a tendency of slightly more for Gpr183^–/–^ compared to WT mice (Fig. 2E) and a similar decrease in lean mass (Fig. 2F). Male WT mice increased more in fat mass compared to male Gpr183^–/–^ mice (Fig. 2G). Male WT mice had significantly higher lean mass at the study start than male Gpr183^–/–^ mice, but both groups showed a stable lean mass throughout the study (Fig. 2H). These data show a sex-specific role of Gpr183 in weight development and body composition during the HFSD.

To gain further insight into the metabolic phenotype of Gpr183^–/–^ mice, we measured fasting blood glucose (fBG) and insulin secretion in both sexes before and after HFSD. Irrespective of the presence (WT) or absence of Gpr183 (^–/–^), fBG levels were stable after 15 weeks of HFSD in all female mice (Fig. 2I). This was likely due to increased fasting insulin levels after HFSD in both female groups (Fig. 2J), indicating compensatory increased insulin secretion in response to energy-rich HFSD-induced insulin resistance. Male WT mice significantly increased their fBG after 15 weeks of HFSD, whereas no difference was observed in male Gpr183^–/–^ mice (Fig. 2K). Although insulin levels in WT male mice significantly increased after HFSD, these mice failed to compensate. In contrast, male Gpr183^–/–^ mice that also showed significantly higher insulin levels after HFSD were able to compensate for insulin resistance (Fig. 2K and 2L). This suggests that Gpr183 has no central role in insulin resistance in female mice, while male mice benefit from the lack of Gpr183.

### Female Gpr183^–/–^ Mice Have an Increased Inflammatory State in Visceral Fat

**Figure 3.**
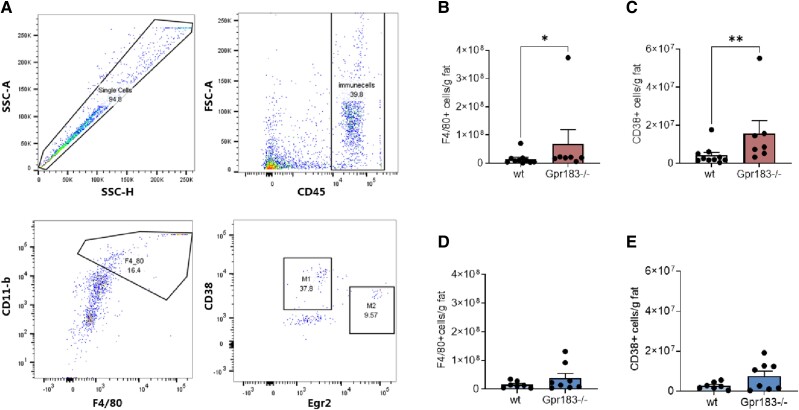
Flow cytometry single-cell analysis of visceral adipose tissue. A, Gating profile for all groups using FlowJo. B, Female overall murine macrophages (F4/80+, cd11b+). C, Female M1 macrophages (CD38+, Egr2−). D, Male overall murine macrophages (F4/80+, cd11b+). E, Male M1 macrophages (CD38+, Egr2−). All animals were age 29 weeks. Number of female mice: wild-type (WT) n = 10 and Gpr183^–/–^ n = 7. Number of male mice: WT n = 7 and Gpr183^–/–^ n = 8. Statistical differences were calculated using the Mann-Whitney test for nonparametric distributions. All data shown as mean ± SEM. *P less than .05; **P less than .01.

We next investigated macrophage infiltration in VAT by flow cytometry (Fig. 3A). Female Gpr183^–/–^ mice had significantly more macrophage (F4/80+, cd11b+) infiltration than WT (Fig. 3B). When we investigated the amount of proinflammatory M1 macrophages (CD8+), the Gpr183^–/–^ females also had significantly more of these cells than WT (Fig. 3C). In contrast, male Gpr183^–/–^ mice had no difference in overall macrophages or M1 macrophages after 17 weeks of HFSD (Fig. 3D and 3E) compared to male WT mice, suggesting that GPR183 contributes to maintaining an anti-inflammatory state in VAT in female mice but not in male mice.

### Reduced Inflammation and Macrophage Markers in Gpr183^–/–^ Livers

**Figure 4.**
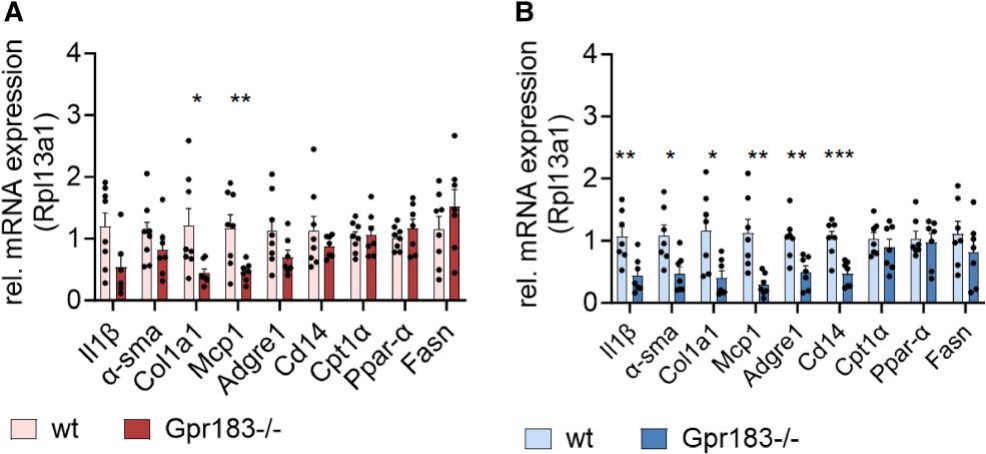
Hepatic gene expression. A, Female hepatic gene expression of selected inflammatory, macrophage, and liver necrosis markers. B, Male hepatic gene expression of selected inflammatory, macrophage, and liver necrosis markers. All animals were age 29 weeks, number of female mice: wild-type (WT) n = 8 and Gpr183^–/–^ n = 7. Number of male mice: WT n = 7 and Gpr183^–/–^ n = 8. Statistical differences were calculated using unpaired *t* test. All data shown as mean ± SEM. *P less than .05; **P less than .01; ***P less than .001.

The finding of elevated macrophage counts in female Gpr183^–/–^ VAT prompted us to investigate the inflammatory state of the liver. Overall, we found significantly lower messenger RNA levels of specific inflammatory and macrophage markers in Gpr183^–/–^ mouse liver compared to WT in both sexes. Female Gpr183^–/–^ mice showed a trend of overall lower levels with significantly lower levels of liver fibrosis markers Col1a1 and Mcp1 compared to WT females (Fig. 4A), a pattern opposite of that observed in VAT macrophage infiltration (see Fig. 3). The same trend was observed in male Gpr183^–/–^ compared to male WT with significantly lower levels of pro-inflammatory marker Il1β, liver fibrosis markers α-SMA, Col1a1, and Mcp1, and macrophage markers Adgre1 and Cd14 (Fig. 4B). No differences were seen between genotypes or sexes in terms of lipid metabolism genes Cpt1α, Ppar-α, and Fasn. This indicates an improved inflammatory liver status in Gpr183^–/–^ mice compared to WT mice when fed an HFSD, in contrast to our VAT macrophage findings.

### Fecal Immunoglobulin A Is Dependent on Gpr183 in Female Mice

**Figure 5.**
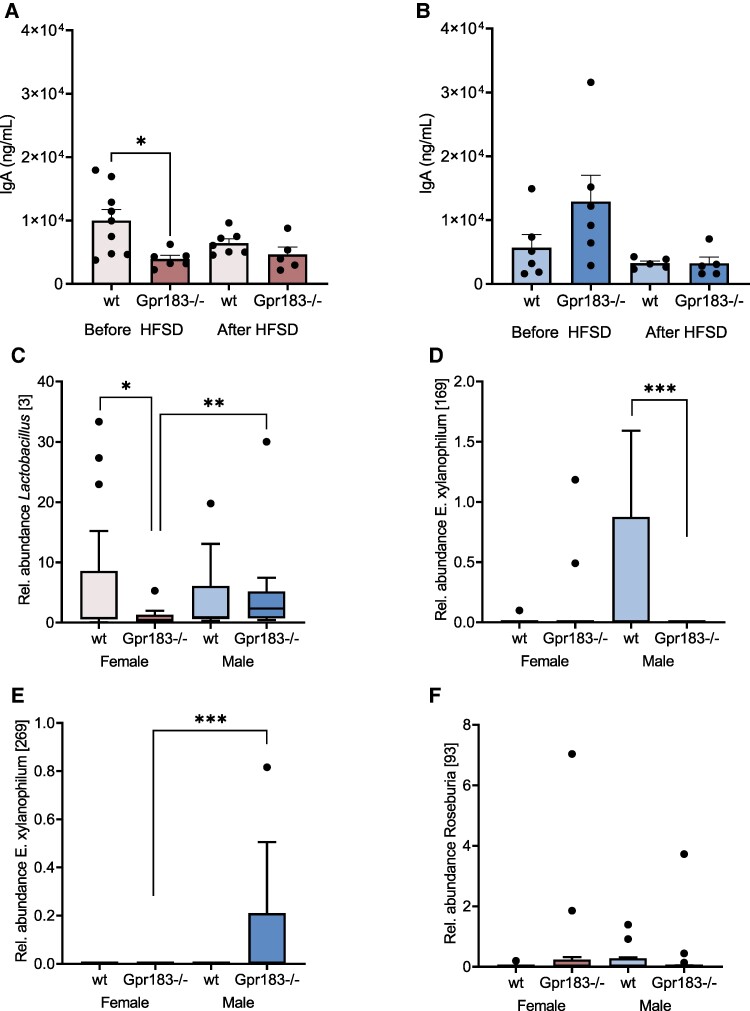
Fecal immunoglobulin A (IgA) content and 16S ribosomal RNA (rRNA) gene-based microbiome analysis. A, Female IgA levels in fecal samples before high-fat, high-sucrose diet (HFSD) (age 11 weeks), wild-type (WT) n = 9 and Gpr183^–/–^ n = 6, and after HFSD (age 27 weeks), WT n = 7 and Gpr183^–/–^ n = 5. B, Male IgA levels in fecal samples before HFSD (age 11 weeks), WT n = 6 Gpr183^–/–^ n = 6 and after HFSD (age 27 weeks), WT n = 5 and Gpr183^–/–^ n = 5. C, Relative abundance for amplicon sequence variants (ASVs) *Lactobacillus* [3] stratified for sex and genotype. D, Relative abundance for ASVs *Eubacterium xylanophilum* group [169] stratified for sex and genotype. E, Relative abundance for ASVs *E xylanophilum* group [269] stratified for sex and genotype. F, Relative abundance for ASVs *Roseburia* [93] stratified for sex and genotype. C to F, All microbiome data were stratified for sex and genotype and not time, so 2 samples per animal are visualized (before and after diet). Number of female WT n = 8 and Gpr183^–/–^ n = 6 and male mice: WT n = 7 and Gpr183^–/–^ = 8 for C to F. For A and B, Statistical differences were calculated using one-way analysis of variance, Tukey multiple comparisons, and data shown as mean ± SEM. For C to F, statistical differences were calculated using negative binomial Wald test with false discovery rate adjustment, and data shown as box and whiskers plot, with Tukey whiskers. *P less than .05; **P less than .01; ***P less than .001.

As stated earlier, the role of Gpr183 in proper migration and maturation of B cells could be important in intestinal homeostasis, as intestinal B1 cells (intestinal plasma cells) are key players in intestinal homeostasis through their production of IgA. We examined the fecal IgA levels in WT and Gpr183^–/–^ mice before and after HFSD. Here, we found that female Gpr183^–/–^ had a lower IgA level before HFSD than WT females, a difference that diminished after diet (Fig. 5A). No difference in fecal IgA was seen before and after HFSD in WT male mice, whereas male Gpr183^–/–^ showed a strong trend (*P* = .067) of decreasing IgA levels after HFSD (Fig. 5B). Taken together, the lack of Gpr183 has more pronounced effects on maintaining IgA levels in female mice.

### Sex-specific Microbiome Associations With Gpr183

Since Gpr183 has been ascribed a central role in the localization and activity of ILC3s in the colonic epithelium [16, 17], we hypothesized that lack of Gpr183 would affect intestinal homeostasis in a proinflammatory direction, possibly through microbiome changes. Therefore, we analyzed the fecal microbiota composition in male and female mice with and without Gpr183. We performed 16S rRNA gene amplicon sequencing of fecal samples before (age 9-11 weeks) and after HFSD (age 19-21 weeks) in all 4 groups of mice. Sequencing reads have been deposited at the NCBI Short Read Archive under BioProject identifier PRJNA1031396 and a heat map can be seen in Supplemental Fig. 2 [30]. First, we investigated differences between groups in alpha diversity: ASV richness and Shannon diversity. While Shannon diversity did not show any statistically significant association with sex, genotype, or HFSD treatment (unadjusted *P* > .18; FDR > 0.8), richness showed a significant association (FDR < 0.05) with genotype. Next, we pooled the data from before and after HFSD and focused on identifying differences in microbial species determined by sex and genotype. Our sex-specific differential abundance analysis between Gpr183^–/–^ and WT mice found that *Lactobacillus* [3] was significantly depleted in female Gpr183^–/–^ mice (Fig. 5C), and 4 other ASVs (*Eubacterium xylanophilum* [169] (Fig. 5D), *Lachnospiraceae* NK4A136 [294], *Lachnospiraceae* NK4A136 [134], and *Ruminococcaceae* UBA1819 [168]) were significantly depleted in male Gpr183^–/–^ mice (DESeq2, FDR < .05). Since the Gpr183^–/–^ female mice specifically had worse metabolic outcomes, we also compared them with Gpr183^–/–^ male mice and identified *Lactobacillus* [3] and *Eubacterium xylanophilum* [269] as depleted in female Gpr183^–/–^ mice (see Fig. 5C and 5E).

We also looked for members of gut-inhabiting *Clostridia* in our data set that could produce the recently identified GPR183 antagonist lauroyl tryptamine [20]. We obtained the 16S rRNA gene sequence of *E rectale* DSM 17629, which has been shown to produce lauroyl tryptamine, and performed a BLAST search to find closely related ASVs in our study. We found 2 ASVs (*Roseburia* [93] and *Roseburia* [376]) to be above 97% similarity, suggesting that they could belong to the same species as *E rectale* DSM 17629 and could be producers of endogenous lauroyl tryptamine. However, these 2 *Roseburia* ASVs exhibited no differential abundance between groups (data not shown). There were an additional 18 ASVs from *Roseburia,* among which *Roseburia* [291] and *Roseburia* [197] were enriched in WT males compared to Gpr183^–/–^ males before and after HFSD treatment, respectively.

### Gpr183 Contributes to Maintaining Glucose Tolerance in Female Mice

**Figure 6.**
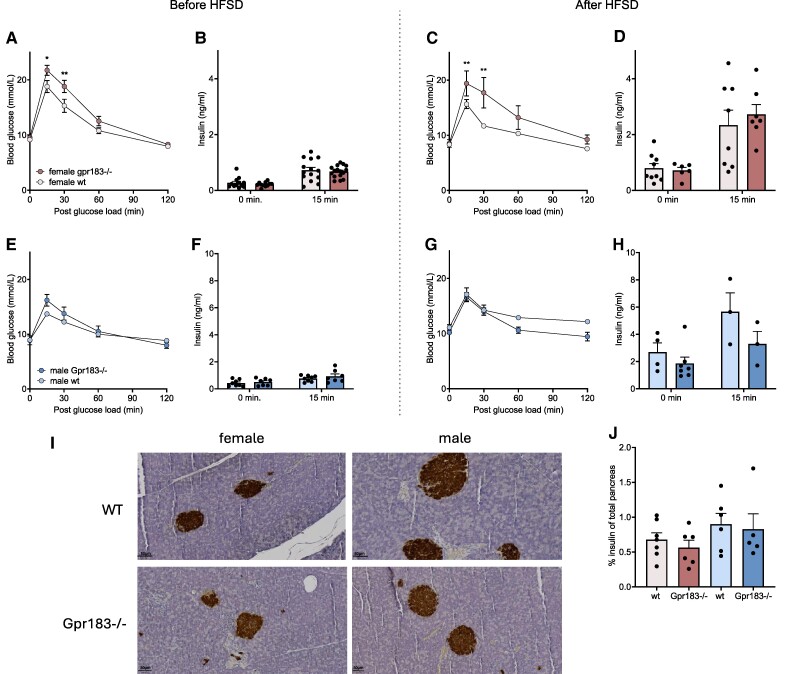
Oral glucose tolerance test (OGTT), before and after high-fat, high-sucrose diet (HFSD) and insulin staining of whole pancreas. A, Female blood glucose levels during OGTT before HFSD, female wild-type (WT) n = 14, Gpr183^–/–^ n = 16. Number of male WT n = 8, Gpr183^–/–^ n = 8 (females from 2 separate studies). B, Insulin levels at time 0 and time 15 minutes, after glucose load, before HFSD. C, Female blood glucose levels during OGTT after HFSD, female WT n = 9, Gpr183^–/–^ n = 7. Number of male WT n = 4, Gpr183^–/–^ n = 8. D, Insulin levels at time 0 and time 15 minutes, after glucose load after HFSD. E, Male blood glucose levels during OGTT before HFSD. F, Insulin levels at time 0 and time 15 minutes, after glucose load, before HFSD. G, Male blood glucose levels during OGTT after HFSD. H, Insulin levels at time 0 and time 15 minutes, after glucose load, after HFSD. I, Immunohistochemical staining of insulin in whole pancreas, by termination after HFSD, scale bar: 50 μm. J, Percentage insulin DAB (diaminobenzidine) staining of total pancreas area, female WT n = 7, Gpr183^–/–^ n = 6. Number of male WT n = 5, Gpr183^–/–^ n = 5. A, C, E, and G, Statistical differences were calculated using 2-way analysis of variance (ANOVA), Tukey multiple comparisons, and B, D, F, and H were calculated 2-way ANOVA mix-effects, I was calculated by 1-way, Tukey multiple comparisons. All data shown as mean ± SEM. *P less than .05; **P less than .01.

Knowing the effect of AT inflammation on glucose homeostasis [21], we investigated the role of Gpr183 in glucose tolerance by performing an oral glucose tolerance test (OGTT) before and after the 15 weeks of HFSD. Female Gpr183^–/–^ mice had an impaired glucose tolerance compared to WT mice before the start of HFSD, with a significantly higher BG at 15 and 30 minutes (Fig. 6A). However, no difference in insulin levels at 15 and 30 minutes was observed between the groups (Fig. 6B). After the HFSD, a lower BG level was seen in all female mice though with a consistently higher BG in Gpr183^–/–^ mice at 15 and 30 minutes compared to WT (Fig. 6C) and with no differences in insulin levels (Fig. 6D). Before and after HFSD, male Gpr183^–/–^ mice showed no difference in BG after an oral glucose load compared to WT mice, and no difference in insulin levels was observed (Fig. 6E-6H). To gain further information regarding insulin secretion, sections of paraffin-embedded whole pancreas were stained with hematoxylin and eosin and subsequently stained for insulin and automatically measured. No differences in morphology or insulin staining were found between the sexes (Fig. 6I and 6J). This suggests that Gpr183 contributes to maintaining glucose tolerance in female mice, regardless of diet.

## Discussion

In this study, we found that Gpr183 contributes to an immunometabolic phenotype with a sexual dimorphism. Regardless of energy content in the diet, *Gpr183* deletion in female mice resulted in an increased macrophage infiltration within the VAT accompanied by a lower fecal IgA level and marked fecal microbiome alterations. In contrast, Gpr183 deficiency in male mice did not affect local macrophage infiltration and fecal IgA levels but resulted in a lowering effect on fBG after HFSD compared to male WT. Gpr183^–/–^ mice showed lower inflammatory gene expression within the liver compared to WT, regardless of sex. An overview of the findings can be found in Table 1.

**Table 1.** Overview of Gpr183^–/–^ metabolic phenotype compared to wild-type before vs after high-fat, high sucrose diet

Outcome	Female Gpr183^–/–^ compared to WT	Male Gpr183^–/–^ compared to WT
**Body weight**	No differences	Smaller
**Fat mass**	No differences	No differences
**Lean mass**	No differences	No differences
**AT macrophage infiltration**	Larger	No differences
**Liver inflammation**	Lower	Lower
**Fecal IgA**	Lower	No differences
**Glucose homeostasis**	Worsen	No differences
**Fasting blood glucose**	No differences	Improved

Abbreviations: AT, adipose tissue; Gpr183^–/–^, Gpr183-deficient; IgA, immunoglobulin A.

Generally, high-fat–fed male mice (C57BL/6J) exhibit larger weight gain, higher fat percentage, lower activity, and worse glucose tolerance than female mice [39]. We therefore anticipated that a metabolic impairment in the Gpr183^–/–^ mice would be strongest in the males. However, even before the intervention, Gpr183^–/–^ male mice (age 10-12 weeks) were significantly smaller in size and had a lower body weight compared to the WT male mice. Intriguingly, a similar phenotype of low preadolescent weight was discovered in male mice lacking CH25H, the enzyme involved in synthesizing the most potent agonist for GPR183, 7α,25-OHC [14]. The same study also described a correlation between body mass index and 25-OHC in males with obesity [14], which underscores sexual dimorphism in oxysterol interactions and indicates a pivotal role of the Gpr183-oxysterol axis in weight regulation from an early age and during weight increase. It is well known that sex hormones influence fat distribution in humans, that is, that women are more prone to weight gain than men, especially during and after menopause, due to changes in estrogen levels [40]. Intriguingly, increased plasma levels of 25-OHC and 27-OHC (preproducts of 7α,25-OHC and 7α,27-OHC) have been described in people with diabetes [12-14], and 7α,25-OH protects human islets against glucotoxicity-induced β-cell damage, as well as potentiate insulin secretion [25]. Adding to this, 25-OHC and 27-OHC act on the estrogen receptor as a full agonist (25-OHC) or partial agonist (27-OHC) [41-43], thereby linking oxysterol ligands for GPR183 with estrogen receptor signaling. Moreover, both 25-OHC and 27-OHC and their enzymes are involved in sexual dimorphism [14, 44, 45]. Accordingly, in high-fat–fed male mice, a decrease of 25- and 27-OHC was seen in AT compared to mice fed a chow diet, while an increase in hepatic levels of 25- and 27-OHC was reported in male db/db mice (leptin receptor mutation) compared to db/lean mice [10]. Together, this could explain the worsening metabolic state in female mice lacking Gpr183 in our study.

Elevated circulating triglycerides serve as additional markers for metabolic dysfunction. However, we were constrained with regard to plasma post termination and could not measure these, which can be seen as a limitation.

GPR183 has also been shown to play diverse roles in bacterial and viral infections with a clear link to metabolism. For example, whole-blood GPR183 expression is downregulated in people with tuberculosis (TB) and additionally type 2 diabetes compared to people with TB alone. Following 6 months of TB treatment, the expression increased, linking low GPR183 expression and metabolic dysfunction with TB severity as supported in Gpr183^–/–^ mice [46]. In contrast, in the lungs of SARS-CoV-2–infected mice, Gpr183 antagonism lowered macrophage infiltration, linking higher Gpr183 activity with SARS-CoV-2 severity [47]. This suggests that oxysterol-GPR183–driven inflammation in the lungs can be attenuated, and that this would be a beneficial therapy against viral respiratory infections [47]. A similar relation has been proposed for GPR183 and Epstein-Barr virus infections [48].

Obesity has been linked with an increased proinflammatory state within the AT [49]. While the infiltration of macrophages in VAT was observed in all mice after 17 weeks of HFSD, a statistically significant increase was seen in female Gpr183^–/–^ compared to female WT, whereas male Gpr183^–/–^ seemed to be protected against this increase when compared to male WT. This is consistent with findings of women being more predisposed to inflammation-associated diseases than men, where differences in the mucosal immune system have been brought forward to contribute to a higher baseline activation in women than men [50].

Inflammation within the AT and increased gut permeability affect the recruitment and activation of immune cells in the liver through cytokine secretion and circulating immune cells (eg, B cells), possibly leading to profibrotic processes within the liver (49-51). VAT from obese mice transplanted into lean mice resulted in increased macrophage liver infiltration in recipient mice compared to mice receiving VAT from lean mice [51]. Gpr183^–/–^ mice showed reduced liver inflammation compared to WT mice that was more pronounced in male Gpr183^–/–^ compared to female Gpr183^–/–^ (Supplemental Fig. 3 [30]). These lower levels in Gpr183^–/–^ mice do not correspond to the elevated VAT macrophage infiltration findings in female and male Gpr183^–/–^ mice compared to WT mice. One possible reason could be that VAT macrophages were a single-cell measurement, whereas liver data were measured on gene-expression level. Furthermore, we know that B and T cells have an important role during proinflammatory immune cell activation in the liver, and Gpr183^–/–^ mice possibly have dysfunctional B and T cells, which might explain a lower level of inflammation within their liver and, thereby, a less pronounced liver phenotype compared to WT, when fed an HFSD.

Moreover, increasing evidence suggests that obesity and its comorbidities can derive from compromised intestinal barrier function [52-54]. GPR183 is expressed on ILC3s and is important in organizing colonic lymphoid structures. ILC3s are important for intestinal barrier function through interleukin-22 and T cell–dependent colonic IgA secretion [16, 55]. GPR183 contributes to intestinal homeostasis through correct B-cell activation and migration through the lymphatic systems before entering lamina propria as IgA-producing B1 cells (intestinal plasma cells) [56, 57]. A previous study concluded that on increased intestinal permeability, through oral indomethacin treatment (10 mg/kg/day), Gpr183^–/–^ mice had significantly higher amounts of B1 cells compared to WT within the peritoneal cavity in female mice [19]. Since we do not know if the B1 cell production of IgA in Gpr183^–/–^ is intact, it is difficult to conclude the mechanism leading to lower IgA levels in Gpr183^–/–^ mice but disabled migration from the peritoneal cavity to the intestines could be a possibility.

Nonetheless, IgA production indicates healthy intestinal homeostasis and low levels are linked to insulin resistance by increased intestinal permeability and downstream inflammation in VAT [23]. Emgård et al [16] found a 50% reduction in colonic lymphoid structures (called colonic patches) but no differences in small intestinal lymphoid structures in Gpr183^–/–^ mice compared to WT. This lower number of colonic patches could be associated with the lower fecal IgA findings in our study, which support the notion that Gpr183 is important for intestinal IgA production, thereby maintaining intestinal homeostasis via antibacterial defense in female mice. Intriguingly, recent findings showed that selective knockdown of Gpr183 in intestinal plasma cells resulted in increased IgA secretion in mice [58]. This suggests a role of Gpr183 in the interplay between intestinally located and circulating immune cells, accordingly, an antagonistic approach has already been raised with evidence in inflammatory bowel diseases and rheumatoid arthritis to impair autoimmune responses within the intestines [59, 60]. In the microbiome, we identified several ASVs depleted in female Gpr183^–/–^ (*Lactobacillus, E xylanophilum [169] and [269]*) compared to both female counterparts and male Gpr183^–/–^ mice. A recent paper discovered a fatty acid amid lauroyl tryptamine, produced by members of Clostridia class, including *E rectale*, to act as an antagonist toward GPR183 and found it in human fecal samples [20]. We searched for *E rectale* in our mice and found 2 closely related ASVs from the *Roseburia* genus (*Roseburia* [93] and *Roseburia* [376]) above the species-level identity threshold of 97%. However, *Roseburia* [93] and *Roseburia* [376] were found in very low abundance in all groups and exhibited moderate differences between Gpr183^–/–^ and WT male mice only after HFSD treatment. As we did not measure lauroyl tryptamine in our study, we do not know if this factor, or other metabolites, contributes to the metabolic phonotypes identified in the absence of Gpr183.

A general consequence of excessive weight gain and adipose tissue inflammation is impaired glucose tolerance. In our study, fasting- and glucose-stimulated insulin levels increased in all groups in response to HFSD but remained similar between the genotypes. In male WT mice, an expected compensatory effect toward HFSD was observed in a statistically significant increase in fBG compared to before the diet, where Gpr183 showed no significant difference before and after HFSD. Challenged with glucose, female mice lacking Gpr183 displayed worse glucose tolerance, irrespective of diet. Fasting or glucose-stimulated insulin levels were increased by diet but were not affected by the genotype of the female mice. We have previously performed an insulin tolerance test on our female Gpr183^–/–^ mice (data not shown), but no differences were found, so we chose to exclude insulin tolerance test from this study setup to decrease stress.

A possible limitation of this study is the timing of the diet given to the mice. An earlier introduction, at around age 6 weeks, could have strengthened our results, particularly regarding the glucose tolerance data. Furthermore, the inclusion of a control group fed a chow diet would have been a strength, especially for comparison to the inflammatory state between Gpr183^–/–^ mice with and without HFSD, as HFSD has a strong effect on low-grade liver inflammation [61]. Since we group-housed our mice for social and ethical reasons, individual food monitoring was not possible, which can also be seen as a limitation.

In conclusion, our study places Gpr183 as a player in intestinal homeostasis with lower levels of the mucosa protecting intestinal IgA in Gpr183^–/–^ mice, most pronounced in female mice. Gpr183 deficiency causes a metabolic sexually diverse phenotype with a proinflammatory state in VAT in female mice, possibly leading to impaired glucose tolerance [59, 60]. As stated earlier, antagonistic approaches have been discussed for GPR183 in inflammatory bowel diseases [59], rheumatoid arthritis [60], and in viral infections [47], and several GPR183 targeting compounds have already been developed [59, 60, 62-64]. Our study highlights that future therapeutic targeting of GPR183 requires inclusion of both sexes, both in preclinical and in clinical studies.

## Data Availability

Original data generated and analyzed during this study are included in this published article or in the data repositories listed in “References.”

## Abbreviations

ASV: amplicon sequence variants
AT: adipose tissue
BG: blood glucose
ELISA: enzyme-linked immunosorbent assay
fBG: fasting blood glucose
FDR: false discovery rate
GPCR: G protein–coupled receptor
Gpr183^–/–^: Gpr183-deficient
HFSD: high-fat, high-sucrose diet
iAUC: incremental area under the curve
IgA: immunoglobulin A
ILC3s: group 3 innate lymphoid cells
OGTT: oral glucose tolerance test
OHC: hydroxycholesterol
PCR: polymerase chain reaction
rRNA: ribosomal RNA
TB: tuberculosis
VAT: visceral adipose tissue
WT: wild-type
